# The *Un Oeuf* study: Design, methods and baseline data from a cluster randomised controlled trial to increase child egg consumption in Burkina Faso

**DOI:** 10.1111/mcn.13069

**Published:** 2020-08-08

**Authors:** Heather Stark, Anteneh Omer, Aïssata Wereme N'Diaye, Amanda C. Sapp, Emily V. Moore, Sarah L. McKune

**Affiliations:** ^1^ Department of Environment and Global Health, College of Public Health and Health Practices University of Florida Gainesville Florida USA; ^2^ School of Nutrition, Food Science and Technology Hawassa University Hawassa Ethiopia; ^3^ Institut de l'Environnement et de Recherches Agricoles, Kamboinsé Agricultural Environnemental Research and Training Center Ouagadougou PO box 476 Burkina Faso

**Keywords:** animal source food, cluster‐randomised controlled trail, complementary feeding, early growth, infant and child nutrition, low‐income countries, nutrition education

## Abstract

In many low‐income countries, such as Burkina Faso, rates of malnutrition are high among children. Research indicates that animal source foods may provide important elements to improve growth and development of young children, especially during periods of rapid development, such as the first 1,000 days of life. The *Un Oeuf* study is designed to test an innovative behaviour change communication strategy to increase egg consumption in children 6–24 months in Burkina Faso, thereby improving dietary diversity and nutritional outcomes. This 1‐year cluster randomised controlled trial tests whether the gifting of chickens by a community champion directly to a child, combined with a behaviour change package of integrated poultry management and human nutrition trainings, can significantly increase egg consumption among children under 2 years in rural communities where egg consumption is very low. The nutrition‐sensitive behaviour change package is designed to increase egg consumption through improving livestock production, women's empowerment and food security at the household level. This paper presents a detailed account of the study design and protocol for the *Un Oeuf* study, alongside a description of the study population. Baseline data show a study population with high rates of malnutrition (stunting 21.6%, wasting 10.8% and underweight 20.4%) and a very low rate of egg consumption—less than 10% among children. Although poultry production is quite common, egg consumption is low reportedly because of cultural norms, including widespread practice of allowing eggs to hatch and a lack of knowledge about the nutritional benefits of egg consumption.

**Key messages**

- Studies have shown the importance of animal source food (ASF) in child diet, especially during periods of rapid development, such as the first 1,000 days of life.
- Eggs have important potential to improve nutritional outcomes in children and can be introduced into a child's diet at 6 months of age.
- Nutrition‐sensitive livestock projects need to better integrate best practices and innovation to change behaviours around ASF.
- The *Un Oeuf* study will test a nutrition‐sensitive Behaviour Change Communication strategy to increase egg consumption in children ages 6–24 months through improved poultry production, women's empowerment and an innovative strategy of gifting chickens to the child.

## 1 INTRODUCTION

### 1.1 Context

Animal source foods (ASF) are important to the human diet, especially during periods of rapid development, such as the first 1,000 days of life (Bhutta, 2013; Darapheak, Takano, Kizuki, Nakamura, & Seino, 2013; Iannotti, Lutter, Bunn, & Stewart, 2014; Nti & Lartey, 2007). Consumption of ASF during this critical window has been shown to increase a child's growth, nutritional status and cognitive function (Neumann et al., 2003, Black et al, 2013). Moreover, ASF consumption and dietary diversity can also reduce stunting caused by chronic malnutrition (Darapheak et al., 2013; Iannotti et al., 2017; Iannotti, Cunningham, & Ruel, 2009; Drewnowski, 2010; Hetherington, Wiethoelter, Negin, & Mor, 2017). In many poor‐resource settings globally, consumption of ASF by young children and pregnant and breastfeeding women is low (Grace et al., 2018; Herrador et al., 2015; Rogers, 1996). In sub‐Saharan Africa, livestock are typically produced for income, gifting and religious practices, rather than for household consumption of ASF (Scoones, 1992; Kondombo, Nianogo, & Kwakkel, 2003; Schneider & Plotnick, 2010). Significant barriers to ASF consumption exist, including cost, availability, fear of disease, lack of nutritional knowledge and cultural beliefs (Gittelsohn & Vastine, 2003; Schneider & Plotnick, 2010; Sonaiya, 2004). All of these perpetuate the lack of ASF consumption within households, despite its nutritional value.

As a renewable source of ASF, eggs provide essential nutrients and are among the lowest cost sources of protein (Drewnowski, 2010). However, eggs remain underutilised in children's diets in many low‐income countries. In Burkina Faso, rates of malnutrition are high (stunting 27.3%, wasting 7.6% and underweight 21.3%), and rates of egg consumption are among the lowest in the world, with only 1.8% of children age 6–8 months, 5% of children age 9–11 months and 4.9% of children age 6–23 months consuming eggs (Iannotti et al., 2014; INSD, 2010; World Bank, 2016).

In many countries, caregivers have little knowledge about the potential benefits of ASF in children's diets (Colecraft et al., 2006). When eggs are consumed, they are often reserved for males or mixed into food, because of their high cost (Gittelsohn & Vastine, 2003). Because female caregivers play an essential role in improving childhood nutrition (Jin & Iannotti, 2014; Nti & Lartey, 2007), it is critical to involve and train them in infant and young child (IYC) feeding (IYCF) practices, as well as livestock production, if ASF consumption is to increase.

Research indicates that increased education of caregivers can lead to increased consumption of eggs among children (Colecraft et al., 2006; Iannotti et al., 2014; Omer, Mulualem, Classem, Vatanparast, & Whiting, 2018). Behaviour change communication (BCC) has been widely used to improve maternal and child nutrition practices (Zaman, Ashraf, & Martines, 2008). In a study in Ethiopia, where chickens were gifted to children by a religious leader and caregivers were counselled to feed children eggs, child consumption of four or more eggs a week increased from 5% to 70% (Omer et al., 2018).

In Burkina Faso, livestock production is important for income generation and family consumption of ASF (Herrero et al., 2013). Poultry ownership is common, with smallholder poultry production accounting for 70% of all poultry production, although production is primarily for income generation (Schneider & Plotnick, 2010). Gender norms influence livestock decision‐making, and although poultry farming is traditionally *managed* by women (Gelli et al., 2017; Schneider & Plotnick, 2010), poultry are typically owned by men, and women often lack decision‐making power (Herrero et al, 2013). Research indicates that livestock ownership can improve food and nutritional security (Hetherington et al., 2017) and that livestock ownership/co‐ownership by women can increase nutritional outcomes among children in the household (Jin & Iannotti, 2014; Smith, Ramakrishnan, Ndiaye, Haddad, & Martorell, 2003; United Nations International Children's Fund [UNICEF], 2015).

Despite a call from the international research community for better integration of nutrition into agricultural programmes for improved maternal and child health outcomes (Ruel & Alderman, 2013), most livestock programmes have thus far failed to meet the bar of nutrition sensitive agriculture (Ruel, Quisumbing, & Balagamwala, 2018). The *Un Enfant*, *Un Oeuf*, *Par Jour* (One Child, One Egg, Per Day) study, referred to hereafter as *Un Oeuf*, combines evidence‐based best practices for nutrition‐sensitive agriculture—BCC strategies and interventions to improve women's empowerment and livestock production—with an innovative strategy of *gifting livestock to a child* to increase egg consumption among children 6–14 months, thereby improving dietary diversity and nutritional outcomes.

## 2 METHODS

### 2.1 Study objectives and hypothesis

The *Un Oeuf* study aimed to increase egg consumption in children ages 6–24 months through a culturally‐tailored BCC strategy to improve poultry production and empower women (Table 1). The primary outcome of this study is the behaviour change of increased egg consumption among IYC, defined by the number of eggs consumed over the past week as reported by their caregiver. Messaging encouraged women to feed enrolled children an egg every day. Secondary outcomes included household poultry production (number of chickens in household), poultry productivity (household egg production), women's empowerment (decision‐making concerning eggs) and nutritional status (*z*‐scores for wasting, underweight and stunting, derived from anthropometric measurements and child age).

**TABLE 1:** Summary of *Un Oeuf* hypothesised impact pathways

*Un Oeuf* activity	Proximal effect	Distal effect
Increase ASF production	• Improve poultry production practices to increase productivity of SHPF • Provide sustainable nutrition and agriculture education • Increase quality and quantity of available ASF, particularly poultry and eggs	• Increase poultry and chicken egg production • Increase quality and quantity of available ASF, particularly poultry and eggs
Increase ASF consumption	• Improve nutritional knowledge • Promote egg consumption among young children through INA training sessions • Address barriers to egg consumption	• Increase egg consumption among children younger than 5 years • Improve nutrition children younger than 5 years
Build resiliency	• Increase livelihood and resilience by improving household nutrition, increasing food security and increasing poultry flock production	• Improve household food security
Reduce poultry disease	• Improve knowledge of poultry housing • Reduce poultry morbidity and mortality • Increase vaccination rates among poultry • Link SHPF with animal/veterinary services • Improve WASH practices	• Improved poultry health and production • Reduce zoonotic disease spread associated with poultry
Promote gender equality	• Employ cross‐cutting themes of gender and nutrition through INA trainings • Engage women in poultry flock management • Train female caregivers in chicken husbandry • Educate women on IYCF practices • Use socio‐ecological model to develop BCC package that influences community and HH level support for women's decision‐making	• Empower women with decision making and poultry husbandry management skills

*Note*: Outcomes—a change in knowledge, attitudes, skill and practices among users. Proximal effects—a direct effect. Distal effects—an effect via a number of intermediary causes.

Abbreviations: ASF, animal source food; BCC, behaviour change communication; HH, household; INA, integrated nutrition and agriculture; IYCF, infant and young child feeding; SHPF, smallholder poultry farm; WASH, water, sanitation and hygiene.

### 2.2 Hypothesis

The *Un Oeuf* study will test the hypothesis that the gifting of chickens by a community champion to a young child combined with a BCC package targeting their female caregivers will increase poultry production, women's empowerment and egg consumption among IYC in Burkina Faso.

### 2.3 Pathways and principles

The study's theoretical underpinnings were guided by UNICEF's Framework of Determinants of Child Undernutrition and Feed the Future's Conceptual Pathways linking livestock, nutrition and women's empowerment (Herforth & Harris, 2014; UNICEF, 2015). These conceptual tools guided the design of a nutrition‐sensitive agriculture BCC package to improve IYC nutrition through increased household production of high‐value foods and decision‐making among women. Using the Social–ecological model to influence and foster change at the community, household and individual levels, the project aimed to empower women's decision‐making in areas that influenced child feeding. The study team engaged local partners to identify community champions, including local elders and community development agents (CVDs). These champions worked with targeted communities to explain the project and garner the support of fathers of targeted children. Participating families had to buy in, literally, to the project, by either gifting the child a chicken from their family flock or purchasing a chicken for the child. The child received three additional chickens from the community champions during a formal gifting ceremony, thereby establishing a flock of four chickens for the child, as well as household and community level engagement and support for the intervention. Women then received a culturally‐tailored BCC package that included monthly group trainings on child nutrition and poultry production, as well as individual‐level counselling. These two primary elements of the BCC package were complemented by a suite of tools and strategies including information, education and communication (IEC) tools and social support. Although the intervention targeted female caregivers' behaviour, males in the households were invited to attend training sessions and to participate in chicken gifting ceremonies to ensure the women had household support. Research findings will inform the design of nutritional education programmes as well as livestock programmes that aim to effect nutritional outcomes.

### 2.4 Study design

The *Un Oeuf* study was a 1‐year cluster randomised controlled trial (CRCT) designed to test whether and to what extent the innovative strategy of community champions gifting chickens to children can improve egg consumption when coupled with a nutrition and agriculture BCC package. The study had three treatment arms: full intervention, partial intervention and control.

- Full intervention arm: children were gifted three chickens from a community champion and a fourth chicken from their family; caregivers received the BCC package.
- Partial intervention arm: received only the BCC package.
- Control arm: received no intervention.

In order to minimise information transfer across treatment arms (Cook, Delong, Murray, Vollmer, & Heagerty, 2016), randomization occurred at the village level. The study design allows examination of the degree to which the BCC package plus the gifting of chickens affects egg consumption (comparing full intervention arm and control arm), as well as the degree to which the BCC package, without any additional assets, can affect egg consumption (comparing partial intervention arm and control arm) among enrolled IYC.

### 2.5 Study setting

**FIGURE 1:**
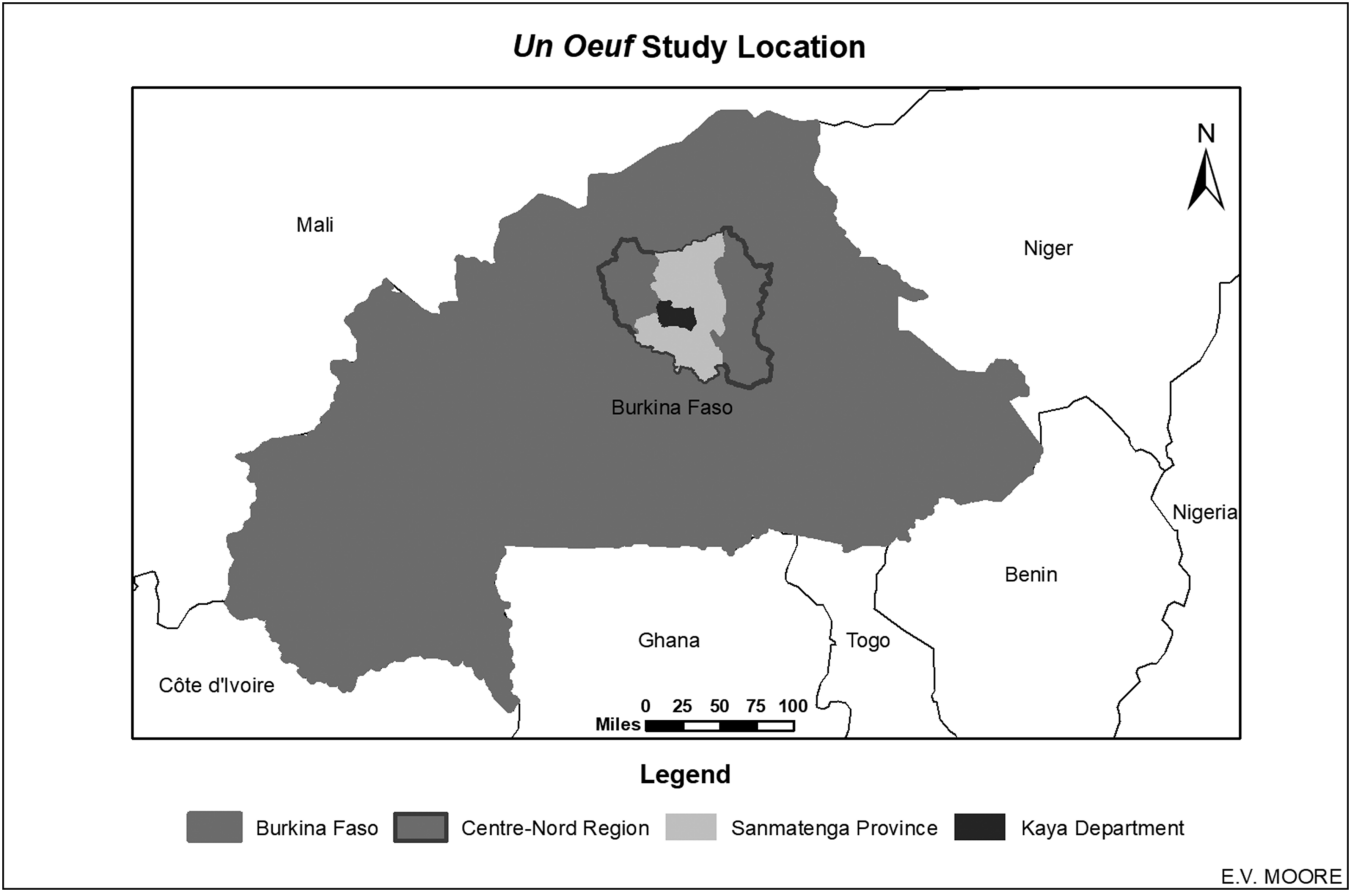
*Un Oeuf* study location

Centre‐Nord Region of Burkina Faso has one of the highest rates of stunting and wasting in the country (INSD, 2010). This region is located in the dry, Sahelian zone and relies primarily on livestock production and limited staple crop production for food security and livelihood. This research was conducted in 18 rural villages within the Kaya Department of the Sanmatenga Province in Centre‐Nord Region (Figure 1). Selected villages were put into treatment arms prior to baseline data collection. The selected rural villages represent a vulnerable population because of high rates of malnutrition, lack of food security and poverty—coupled with broader challenges of climate change, political instability, violence and conflict.

### 2.6 Study participants' inclusion and exclusion criteria

This study randomly targeted recruitment of participants from the total population of children 6–12 months old living in selected villages. Children with a history of egg allergies or severe malnutrition were excluded from the study. If children were identified from within the same household, only one child, randomly selected from among otherwise eligible children in the household, was included. A full description is provided in Section 3 according to CONSORT recommended guidelines (Schulz, Altman, & Moher, 2010).

### 2.7 Sample size and power

With assistance from the Clinical and Translational Science Institute at the University of Florida, a power analysis for the CRCT was conducted, modelled on differences in egg consumption found in the preliminary analysis of Omer's study in Ethiopia (Omer et al., 2018). The power analysis was based on children consuming four or more eggs a week and accounted for a cluster design. The proportion of children eating four or more eggs per week in the full intervention arm was assumed to be 1% under the null hypothesis and 13% or larger under the alternative hypothesis. A sample size of 270 children (90 per intervention arm) was determined on the basis of its ability to detect a difference between full intervention and control arms of 13% with a two‐sided *z* test (unpooled) with a *P* value of 0.05 and a power of 80%. This sample size assumes an intracluster correlation of 0.02 and a design effect of 1.28.

### 2.8 Detailed study intervention description

See Data S1 for the project timeline.

### 2.9 Gifting of chickens

Children in the full intervention arm were gifted three egg‐laying local chickens by community champions. Community champions were village chiefs and community leaders, including CVDs and community health educators, identified by the research team through formative research as most appropriate for catalysing change. This element of the study aimed to leverage existing sociocultural spaces to increase uptake of nutrition messaging and behavioural adherence. Families of participating children were asked to match the three gifted chickens with one additional chicken, purchased or gifted, to ensure household buy‐in. Community champions reinforced messages that eggs produced from the gifted chickens belonged to the enrolled child and were for their consumption alone, underscoring the importance of ASF during this critical period of child growth and development.

### 2.10 BCC package

The BCC package consisted of monthly integrated nutrition and agriculture (INA) trainings, monthly one‐on‐one counselling of caregivers and messaging to reinforce key behaviours. A detailed description of these components is found below.

#### 2.10.1 INA trainings

Using a One Health, training‐of‐trainers model, INA trainings were implemented by local agriculture extension workers (AEWs) and community health workers (CHWs). The interdisciplinary teams of INA trainers received three 2‐day trainings from expert consultants in agriculture and nutrition. INA trainings occurred monthly for 10 months in each of the intervention villages (*n* = 12) and were conducted collaboratively by teams of CHWs and AEWs.

Ten monthly INA training sessions were provided to the child's caregiver in the full and partial intervention groups reinforcing key messaging including the importance of ASF consumption, proper poultry husbandry procedures, water, sanitation and hygiene (WASH) practices and zoonotic disease prevention. Caregivers received hands‐on training on how to hard boil, smash and feed the egg, in its entirety, to an infant or young child. Techniques, such as mixing the egg with extracted breast milk or using an inverted spoon to mimic a nipple in the child's mouth, were included in early trainings to facilitate feeding 6–7‐month‐olds. Given sociocultural norms of food sharing and concerns about the acceptability and willingness of caregivers to give only the enrolled child an egg while other children were in the household, INA trainings stressed the value of ASF consumption at critical ages and encouraged improved management of *other* household chickens for provision of eggs to other children.

Families were provided with informational demonstrations regarding proper chicken housing in order to reduce the spread of zoonotic diseases and increase poultry productivity. The importance and potential of closed poultry systems were discussed and demonstrated, although no housing was constructed through the project. Trainings aimed to empower female caregivers through lessons on poultry husbandry practices and vaccination demonstrations. Nonenrolled women in the community and men were invited to participate in the training sessions and activities. In addition, INA trainings served as a space for interaction between caregivers to share their experiences and learning about poultry husbandry and production, egg feeding and other related issues.

#### 2.10.2 One‐on‐one counselling with behavioural change flipbook

An illustrated flipbook was created using SPRING's IYCF images (The Spring Project, n.d.) and guided by UNICEF Community IYCF Counseling Package emphasizing key messages taught in monthly INA trainings (Data S2). Flipbooks were designed for an illiterate population, using culturally‐appropriate pictures to convey important messages, and distributed to all caregivers enrolled in the full and partial intervention groups. Caregivers brought the flipbooks to each monthly monitoring visit, and enumerators used them during the one‐on‐one counselling to adjust behaviours, clarify misunderstandings and better understand constraints. As such, caregivers interacted with project staff at least twice per month, once through individual counselling at data collection and once through group INA training.

#### 2.10.3 Behaviour change messaging

During INA trainings, project staff facilitated development of a jingle, ‘One child, one egg, each day’, which women adopted and sang enthusiastically at each monthly meeting. In addition, T‐shirts, initially developed for data collectors and the project team, were extremely well received in the targeted communities and thus were ultimately developed and used as educational materials across the project. The T‐shirts depict the same message, using images from the flipbook, widely visible and understandable, independent of literacy.At the end of the study, all caregivers were invited to a closing ceremony. Women in the intervention groups were encouraged to serve as the *trainers*, providing brief essential information on the importance of ASF consumption and the potential benefits of eggs in child development to caregivers from the control arm of the study. During the closing ceremony, the study provided two chickens to each caregiver in the partial intervention and control arms.

### 2.11 Data collection and storage

A household survey (HHS) was conducted monthly, with comprehensive HHS conducted at baseline, midline and endline and shorter monitoring surveys conducted in between. Three graduate students in Burkina Faso were trained to conduct all data collection, coordinated and overseen by a local project manager. Data were collected using Research Electronic Data Capture (REDCap) (Harris et al., 2009) software on Samsung tablets encrypted for data collection in accordance with Institutional Review Board policies.

#### 2.11.1 Household survey

The HHS included modules on household demographics; child health and feeding practices; caregiver knowledge, attitudes and practices surrounding egg consumption and dietary diversity; livestock and poultry practices; WASH; and household decision‐making. Dietary assessment methods included a 24‐h dietary recall for both child and caregiver in which caregivers were asked what foods they had consumed in the past 24 h. Subsequently, caregivers were then asked if any items from a list of local foods were consumed.

#### 2.11.2 Monthly monitoring survey

Caregiver/child dyads were monitored for 10 months to capture child egg consumption and dietary diversity. During the monthly surveys, caregivers were asked if their child had consumed certain foods over the past week. If they reported child egg consumption, they were asked additional questions regarding quantity and frequency of eggs consumed. Egg production and flock size were also monitored monthly.

#### 2.11.3 Anthropometric measurements

Anthropometric measurements (weight and length) were collected monthly for enrolled children and included weight to the nearest 0.1 kg (SECA model 874 portable scale) and recumbent length to the nearest 0.1 cm (UNICEF height board). Measurements were repeated three times to assure accuracy; the median was used in analysis. The TARE setting was used to determine the child's weight while being held by his/her caregiver. Training on growth monitoring was provided to data collectors by CHWs.

### 2.12 Ethical considerations

The protocol, site‐specific informed consent forms (French and English versions), participant education and recruitment materials, and other requested documents were reviewed and approved by ethical review bodies at both the University of Florida (UF) (IRB201702810) and the Committee of Ethics in Burkina Faso prior to starting this study. This study is registered with Clinical Trials.gov: NCT04135625. Verbal informed consent was obtained from each child's caregiver prior to enrolment, and participants were allowed withdraw at any time without consequence.

### 2.13 Data analysis

This study carried out an intent‐to‐treat approach, which included attempts to track participants through follow‐up in the longitudinal study for 10 months. Missing data were removed using complete case analysis for cases missing completely at random (MCAR), and analysis was conducted on the population size (*n* = 260). For branching variables, analysis was only conducted on responses from participants to whom the question applied, thus reducing the sample size in certain analyses. All data collection, extraction and cleaning were conducted in Excel and REDCap, whereas summary statistics, tables and analyses were generated in R statistical software version 3.6.0 (R Core Team, 2019). Anthropometric *z*‐score calculations were generated in R software using the World Health Organization (WHO) Child Growth Standards (WHO, 2006).

Analysis of data in the longitudinal CRCT will include bivariate analysis and, where statistically indicated, mixed‐effects predictive modelling of four key outcomes: behaviour change, women's empowerment, poultry production and growth of children over time. Mixed‐effects models will use covariates as fixed effects, whereas the 18 clustered villages built into this study design will be used as random effects. Treatment estimates will be generated by controlling for baseline variables within the mixed models. Statistical significance for all analyses will be set at 5%, with all statistical testing being two‐sided.

## 3 RESULTS

Of the 77 villages within Kaya district, seven were eliminated because of their urban designation. On the basis of population data for the remaining 70 villages, a population increment for selection of 18 clusters was calculated (3,892). From a list of villages with a running population total, 18 villages were selected, beginning from a randomly generated number (59,413, generated 13 May 2018 on numbergenerator.org). This approach ensured smaller villages had the same likelihood of being selected as larger villages. From the 18 selected villages, six villages were randomly assigned to each of the three intervention arms (full, partial and control) by drawing the village name from a box and sequentially assigning it to an intervention arm. Post hoc assessment of village treatment arm assignment was conducted to determine and, if necessary, minimise the potential impact of villages within a treatment arm having better access to a natural water resource. No modifications to the villages' randomized assignment were necessary.

**FIGURE 2:**
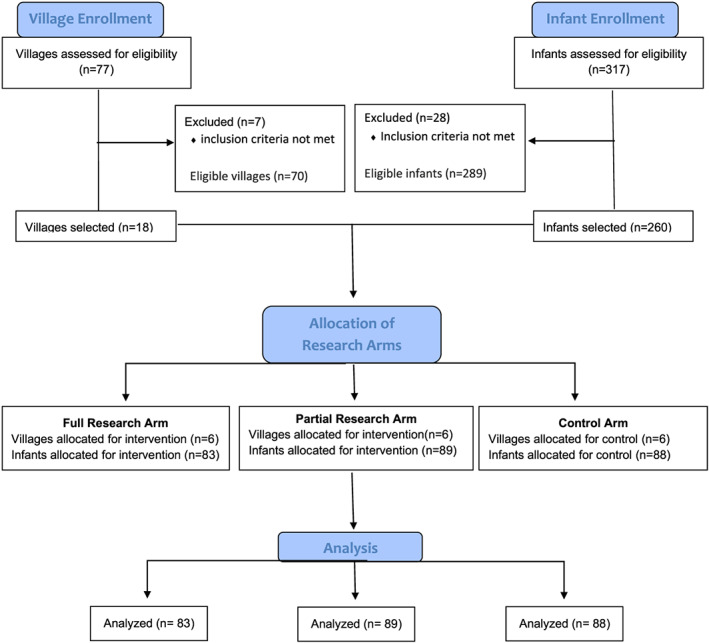
Flow diagram

Although the study aimed to enrol children age 6–12 months, there were not enough available children within this age range in most villages. Consequently, it was determined that expanding the age of enrolment in villages that had less than the necessary 15 eligible children would not jeopardise the objective of the study; thus, children between the ages of 4 and 18 months were enumerated. A total of 317 children were enumerated in the 18 villages during June 2018 with the assistance of CVDs. Of those 317 enumerated children, 289 were identified as eligible. In villages where the number of eligible children was greater than 15, children from the same households were excluded (28 children excluded for this reason), and 15 eligible children were randomly selected from each village. In villages that had 15 or fewer eligible children, all eligible children were selected for enrolment, including those from the same household. This occurred in five communities—three in the full intervention arm and one in the partial intervention and control arms. Screening for malnutrition and egg allergies was conducted by local CHW familiar with each child; no child was found to have severe malnutrition or history of egg allergy at the time of enumeration or consent; thus, no exclusions occurred for these reasons. Ultimately, 260 children were consented and enrolled in the study. Village selection, enumeration, recruitment, enrolment and intervention details are shown in Figure 2.

### 3.1 Baseline characteristics

Baseline characteristics are derived from analysis of 260 dyads enrolled in the study as presented in Table 2. Caregivers were mothers of the child with one exception (grandmother); thus, mother/caregiver is used interchangeably. At baseline, mean age of children enrolled was 9.87 months; mothers' mean age was 27 years. The majority of mothers had received no formal education and were illiterate. The median household size was 14.5 people (range 3–80) with an average of three children under the age of five, most commonly led by a male head of household. Polygamy is common within our study population, with half of the women indicating being married to men who have more than one wife. Participating women were primarily first (65.4%) and second (21.2%) wives, with a range of first to fifth wives. Women described themselves as being Muslim (76.2%) or Christian (23.8%). Most households reported crop production and animal husbandry as a primary source of livelihood; fewer reported trading of goods or panning gold. In terms of sanitation, almost all households had access to an improved water source, and more than half reported having access to and using an improved latrine. Open defecation was practised by a fourth of respondents.

**TABLE 2:** Baseline characteristics of the *Un Oeuf* study population (*n* = 260)

	Number (percent, unless otherwise noted) [range]
**Child**	
Age in months (SD)	9.87 (3.13)
Sex (female)	127 (48.8%)
**Maternal**	
Age in years (SD)	27.0 (6.60)
Education	
No formal education	207 (79.6%)
Primary education	27 (10.4%)
Secondary education	15 (5.8%)
Koranic school	10 (3.8%)
Maternal literacy	38 (14.6%)
Age first birth, median	18 [13, 25]
Number births	3.46
**Household**	
Household size, median	14.5 [3.00, 80.0]
Head of household	
Husband	208 (79.2%)
Grandfather	53 (20.4%)
Grandmother	1 (0.4%)
Household livelihood	
Crop production	249 (95.8%)
Animal husbandry	144 (55.4%)
Livelihood—other	43 (16.5%)
**Religion**	
Muslim	198 (76.2%)
Christian	62 (23.8%)
**Ethnicity**	
Mossi	245 (94.2%)
Other	15 (5.8%)
**Household livestock ownership**	
Any livestock	249 (95.8%)
Cow	140 (53.8%)
Sheep	189 (72.7%)
Goat	176 (67.7%)
Chicken	212 (81.5%)
Number of chickens, median	3.00 [0.00, 100]
Donkey	185 (71.2%)
**Water and sanitation**	
Drinking source type	
Improved—other	235 (90.4%)
Improved—piped	18 (6.9%)
Unimproved	6 (2.3%)
Latrine	
Improved latrine	172 (66.2%)
Practice open defecation	62 (23.8%)

*Note*: Age in months and years are reported as standard deviations (SD).

### 3.2 Livestock production characteristics and poultry practices

Table 2 describes baseline characteristics of household livestock practices. Livestock ownership in this region is common, with almost all households owning livestock. Chickens were the most common type of livestock owned (81.5%), followed by sheep (72.7%) and donkeys (71.2%). Households owned a median of three (range 0–100) chickens, producing a median of one egg daily (range 0–60). Eggs were kept for the objective of increasing flock size (97.6%), and very few were consumed (0.9%). Chickens were reportedly kept to reproduce and increase flock size, as well as sell, consume and to gift or tithe. Most caregivers (69.8%) reported they did not have enough chicken eggs to feed their child an egg per day. Household chicken husbandry practices varied; about half (55.8%) had a coop, and a quarter (30.8%) had a separate area from their living quarters for chickens to roam during the day. No one reported corralling their chickens all the time. Women reported corralling their chickens sometimes (34%) or never (66%), as chickens in this region primarily free range scavenge for food. At night, 68.4% cooped or enclosed their chickens, 21.9% kept their chickens in their house, and 10.5% kept their chickens in a tree. In this region, chickens scavenge for food. Most households (83.5%) reported that their flocks were vaccinated against Newcastle disease, a disease that commonly kills whole flocks.

Caregivers were also asked about their child's interaction with chicken excrement and if they believed that chicken excrement could cause illness or disease. About one quarter (26.9%) reported their child comes into contact with chicken excreta, with exposure from their and other families' flocks. Most caregivers (81.4%) did not believe that chicken excrement caused illness.

### 3.3 IYCF practices

Data surrounding IYC egg consumption, feeding practices and 24‐h dietary recall are presented in Table 3. All women reported currently breastfeeding on demand at baseline, with exclusive breastfeeding having been practised for an average of 6 months. Most children (85.8%) received complimentary feeding beginning at 6 months. Common complimentary foods included sorghum, rice and millet. Very few (10%) mothers reported ever feeding a child chicken eggs, and even fewer (4.2%) had fed the enrolled child an egg in the past week. More mothers (17.7%) reported ever having fed children guinea fowl eggs. Reasons for not feeding their child chicken eggs included not having enough eggs (48.3%), prioritizing hatching the eggs (28.7%), cost/money (8.4%), lack of knowledge about feeding a child an egg (9.6%) and not wanting to interrupt the chicken's life cycle (1.5%). Among the few mothers who fed children eggs, eggs were acquired at markets, with fewer acquired from their or their neighbours' flocks. Most mothers reported never having received nutritional education about feeding children eggs (79.6%). Importantly, at baseline, over half of women (60%) indicated, given current resources, skills and knowledge, that they thought they could feed their child an egg a day; another 8.1% responded they did not think they could feed their child an egg a day and 32.9% that they did not know. However, most mothers (87.7%) also indicated it would be somewhat to very difficult to obtain the eggs to feed their child an egg per day.

**TABLE 3:** Baseline child feeding practices, diet diversity, women's empowerment and child anthropometry of the *Un Oeuf* study population

Feeding practices	Overall (*n* = 260)
**Child feeding practices**	
Ever feed child chicken eggs	26 (10.0%)
Targeted child ate chicken eggs in the past week	11 (4.2%)
Number of eggs fed to target child in past week, median	0.00 [0.00, 7.00]
**Child dietary diversity score (0–4) food groups consumed**	
Median	1 [1,4]
**Dietary diversity score‐mother (0–5) of food groups consumed**	
Median	1 [1,4]
Decision‐making	Overall (*n* = 253)
**Decision maker surrounding food given to child**	
Self	169 (65%)
Other	84 (32.3%)
**Decision maker surrounding what is done with chicken eggs produced by household chickens**	
Self	83 (31.9%)
Other	169 (65%)
Child anthropometric data	Overall (*n* = 256)
**Nutritional status (median)**	
WLZ (wasting) [SD]	−0.68 [−4.73, 2.13]
WAZ (underweight) [SD]	−1.15 [−4.23, 1.23]
LAZ (stunting) [SD]	−1.16 [−3.82, 1.98]
**% Wasted**	
Moderate wasting (< −2, ≥ −3 SD, %)	15 (5.8%)
Severe wasting (< −3 SD, %)	13 (5.0%)
**% Underweight**	
Moderate underweight (< −2, ≥ −3 SD, %)	39 (15.0%)
Severe underweight (< −3 SD, %)	14 (5.4%)
**% Stunted**	
Moderate stunting (< −2, ≥ −3 SD, %)	41 (15.8%)
Severe stunting (< −3 SD, %)	15 (5.8%)

*Note*: Wasting, underweight and stunting are reported as standard deviations (SD) of the WHO Child Growth Standards median.

Abbreviations: LAZ, length‐for‐age *z*‐score; WAZ, weight‐for‐age *z*‐score; WLZ, weight‐for‐length *z*‐score.

An indicator of dietary diversity was calculated for each child and mother using a 24‐h dietary recall (Table 3). Although the study aimed to calculate minimum dietary diversity (MDD) score for IYC and for women (INDDEX Project, 2018), data on vitamin A source foods and green leafy vegetables were unintentionally omitted from the questionnaire. Consequently, a modified dietary diversity (dd) was calculated for both IYC and for women. On the basis of food groups from the MDD‐IYC, our dd score for IYC is composed of the six food categories: (1) grains, roots and tubers, (2) legumes and nuts, (3) dairy, (4) flesh foods, (5) eggs and (6) fruits and vegetables. Nearly half (46.5%) of children were not receiving any complementary foods at baseline and had a dd score of 0. All IYC consumed breast milk. Formula was consumed by 56.5% overall and by 61% among those who had a dd score of 0. Notably, IYC consuming only formula and breast milk at baseline ranged in age from 5 to 15 months (median, 9 months). Children who had a dd score of 1 (47%) were only consuming grains. A small minority of children (6%) had a dd score of 2, which consisted of consuming grains and either meat (7%) or dairy (3.5%). Our modified dd score for women was calculated on the basis of the eight food categories collected that parallel the MDD‐W: (1) grains, roots and tubers, (2) pulses, (3) nuts and seeds, (4) dairy, (5) meats, poultry and fish, (6) eggs, (7) vegetables and (8) fruits. Most mothers (81.2%) reported a dd score of 1, with grains being the most common food consumed. Fewer women (17.7%) reported a dd score of 2; among these women, grains were supplemented by meat (13%), nuts and legumes (5%), fruits and vegetables (2%) or dairy (2%). Very few (1.2%) reported a score of 3, consuming flesh meat, nuts, fruit and/or pulses.

### 3.4 Anthropometric assessment

Nutritional outcomes are presented in Table 3. Wasting, underweight and stunting were defined by weight‐for‐length *z*‐scores (WLZ), weight‐for‐age *z*‐scores (WAZ) and length‐for‐age *z*‐scores (LAZ) of less that −2, respectively. Severe cases were those with *z*‐scores less than −3. Baseline prevalence rates of wasting, underweight and stunting were 10.8% (median WAZ − 0.680 [−4.73, 2.13]); 20.4% (median WLZ −1.15 [−4.23, 1.23]); and 21.6% (median LAZ score −1.16 [−3.82, 1.98]), respectively. Baseline prevalence rates of severe wasting, underweight and stunting were 5.0%, 5.4% and 5.8%, respectfully.

### 3.5 Women's decisionmaking

Women caregivers were asked about household decisionmaking. More than half reported being the decision maker regarding what foods are given to the child yet, and more than half reported someone else was the decision maker concerning what was done with eggs produced by the household (Table 3).

## 4 DISCUSSION

Findings from analysis of baseline data reinforce those presented in the literature. Undernutrition in the *Un Oeuf* study population is high, with 21.6% of children stunted and 10.8% wasted. Wasting in this study population is higher than in the general population. Like many other regions in sub‐Saharan Africa, baseline findings confirm that egg consumption by children is very low. Resources for diversifying diets are scarce, yet chicken eggs are available. There is significant variation in the number of chicken eggs produced per household in the study population (ranging from 0 to 60, median 1), driven by a large range in the number of poultry owned. Most families have chickens but are not consuming the eggs; rather, they are hatching the eggs. Caregivers of participating children are generally unaware of the nutritional value and benefits of eggs for child development, highlighting the necessity for nutrition‐sensitive education and approaches to agriculture. Only one third of women reported being the decision maker about what is done with eggs, yet a majority make decisions about what foods are given to the child; therefore, increasing women's household decision‐making power may influence egg consumption in child diet.

Limitations to the study design and baseline data presented here include the number of eligible children in each village, errors around date of birth and consequent child age at enrolment, the potential for social contamination and the potential for recall bias.

The research team did not anticipate difficulty finding 15 eligible children in each village. Despite extensive effort to validate child birthdates, four children younger than 6 months were recruited and enrolled into the study—two in the control arm and one in each intervention arm. These children were nearly 6 months old when the intervention started, and messaging was clear in all trainings that consumption of eggs was to commence after 6 months. The study team considered dropping these children from the study, but to preserve sample size, they were retained.

A CRCT was designed to reduce contamination of information between the intervention groups and the control group, although information sharing between study groups may occur.

Finally, the study design relies heavily on mothers' reporting of behaviour and may reveal a number of response biases such as recall bias in reporting past behaviour and social desirability bias, where their answers reflect their own perception of what is good behaviour. Additionally, responses in the control group may reflect a demand bias, where they change their behaviour (or report to) on the basis of participation in data collection activities. To limit these biases, women were prompted to answer detailed questions about egg consumption *only if* they reported feeding their child an egg in the past week when asked a list of food groups fed to their child in the past week. In addition, unannounced visits to the households were conducted to monitor and triangulate flock health, poultry production and productivity, and caregiver/child behaviour.

The results of this research will have important implications for understanding how nutrition‐sensitive culturally tailored BCC strategies can be combined with livestock interventions to increase egg consumption and improve dietary diversity in rural, low‐income settings. Although research to understand the role of ASF consumption and child growth and development is increasing, the evidence base for *how* to increase ASF consumption among vulnerable populations is still quite small. Importantly, this research will test not only the integration of a nutrition‐sensitive, culturally embedded behaviour change package into efforts to increase livestock assets but also the scalability of Omer's innovation—the gifting of chickens directly to children by a community champion. Findings from this study have the potential to influence the approaches of government and nongovernmental organizations to improve the health and nutrition of young children in rural Burkina Faso. In turn, those efforts may inform improvements in other low‐ and middle‐income countries.

## CONTRIBUTIONS

HS, SM, AO and AW conceived of the study. HS, SM, AO and AW initiated the study design, and HS, SM, AO, AW and EM were involved in implementation. EM conducted data cleaning, quality control and analysis support. AS provided statistical expertise in clinical trial design and conducted the primary statistical analysis. HS led the write up of this manuscript, with significant contribution by SM and AO. All authors contributed to the refinement of the study protocol and approved the final manuscript.

## Supporting information


**Data S1.** Annex 1. The Un Oeuf Study Timeline (m = month)

**Data S2.** Annex 2: Images from the behaviour change communication flipbook given to participants in the *Un Oeuf* Study
Figure S1. Cover of laminated flipbook
Figure S2. Illustrates the message to begin feeding your child complementary foods at age 6 months in addition to continuing breastfeeding.
Figure S3. Illustrates the projects key behaviour change message‐ *feed your child one egg per day‐ to improve the heath, growth and development.*
Figure S4. Illustrates the procedure for preparing eggs.
Figure S5. illustrates how to prepare the egg for the child.
Figure S4. Illustrates the procedure for preparing eggs.
Figure S5. illustrates how to prepare the egg for the child.
Figure S6: Illustrates the importance of monitoring a child's health and growth.
Figure S7. Illustrates that four chickens are recommended to produce enough eggs to feed a child an egg a day.
Figure S8. Illustrates the importance of keeping livestock in a separate area than the home environment as well as keeping the home environment clean and free of animal waste in efforts to prevent diseases caused by livestock.
Figure S9. Illustrates the importance of vaccinating poultry to prevent disease and death among livestock

## Data Availability

Data from this project will be stored and available through Harvard Dataverse. This study is registered with Clinical Trials.gov NCT04135625.
