# Supplementary material for: The Un Oeuf study: Design, methods and baseline data from a cluster randomised controlled trial to increase child egg consumption in Burkina Faso
Source: Matern Child Nutr. 2020 Aug 8;17(1):e13069. doi: 10.1111/mcn.13069 (PMC7729644; doi:10.1111/mcn.13069)
Supplement: Supplementary file 1 — Data S1. Annex 1. The Un Oeuf Study Timeline (m = month) [file MCN-17-e13069-s001.pdf]

Annex 1. The Un Oeuf Study Timeline (m = month)

Activity	M1	M2	M3	M4	M5	M6	M7	M8	M9	M10	M11	M12
Formative research and enumeration	■	■										
Consent and enrollment		■										
Baseline survey		■										
Chicken gifting ceremony (full arm)			■									
Monthly monitoring survey			■	■	■	■		■	■	■		
Midline household survey							■					
Endline survey											■	
Anthropometric screening		■	■	■	■	■	■	■	■	■	■	
Training of the trainers		■				■						
INA training sessions (full and partial arms)			■	■	■	■	■	■	■	■	■	■
Monthly counseling (full and partial arms)			■	■	■	■	■	■	■	■	■	■
Monitoring and Evaluation							■					
Closing ceremony/ INA training for all caregivers												■
Distribution of 2 chickens (partial and control arms)												■
