# Supplementary material for: The Un Oeuf study: Design, methods and baseline data from a cluster randomised controlled trial to increase child egg consumption in Burkina Faso
Source: Matern Child Nutr. 2020 Aug 8;17(1):e13069. doi: 10.1111/mcn.13069 (PMC7729644; doi:10.1111/mcn.13069)
Supplement: Supplementary file 2 — Data S2. Annex 2: Images from the behaviour change communication flipbook given to participants in the Un Oeuf Study Figure S1. Cover of laminated flipbook Figure S2. Illustrates the message to begin feeding your child complementary foods at age 6 months in addition to continuing breastfeeding. Figure S3. Illustrates the projects key behaviour change message‐ feed your child one egg per day‐ to improve the heath, growth and development. Figure S4. Illustrates the procedure for preparing eggs. Figure S5. illustrates how to prepare the egg for the child. Figure S4. Illustrates the procedure for preparing eggs. Figure S5. illustrates how to prepare the egg for the child. Figure S6: Illustrates the importance of monitoring a child's health and growth. Figure S7. Illustrates that four chickens are recommended to produce enough eggs to feed a child an egg a day. Figure S8. Illustrates the importance of keeping livestock in a separate area than the home environment as well as keeping the home environment clean and free of animal waste in efforts to prevent diseases caused by livestock. Figure S9. Illustrates the importance of vaccinating poultry to prevent disease and death among livestock [file MCN-17-e13069-s002.pdf]

Annex 2: Images from the behavior change communication flipbook given to participants in the *Un Oeuf* Study

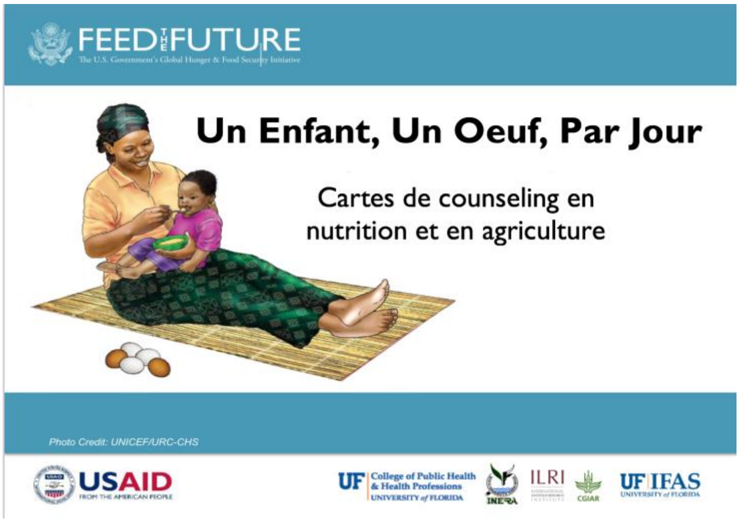


Figure 1. Cover of laminated flipbook

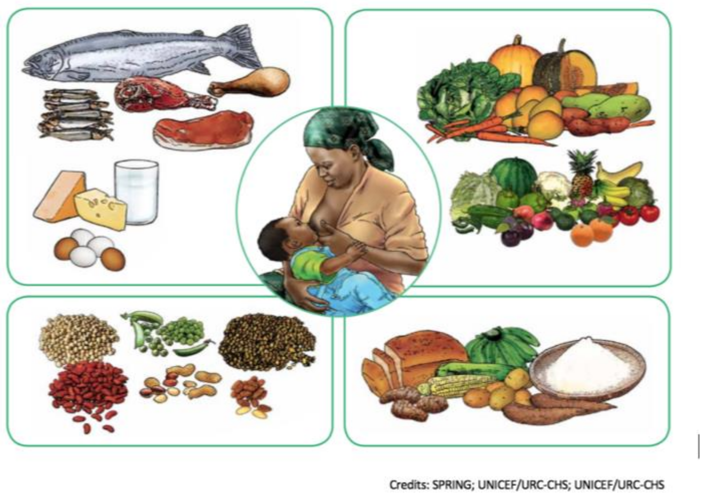


Figure 2. Illustrates the message to begin feeding your child complementary foods at age 6 months in addition to continuing breastfeeding.

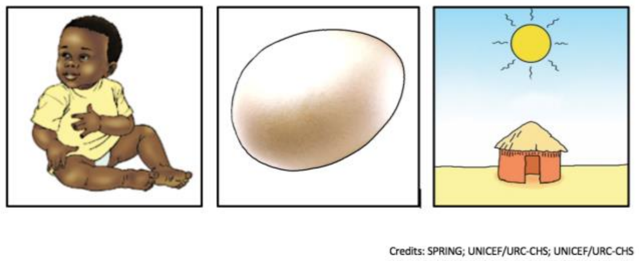


Figure 3. Illustrates the projects key behavior change message- *feed your child one egg per day- to improve the heath, growth and development.*

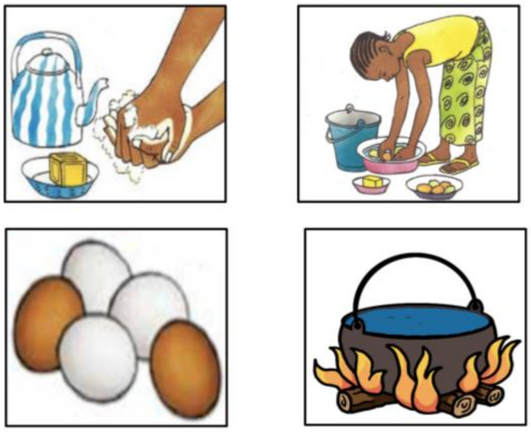

Figure 4. Illustrates the procedure for preparing eggs.

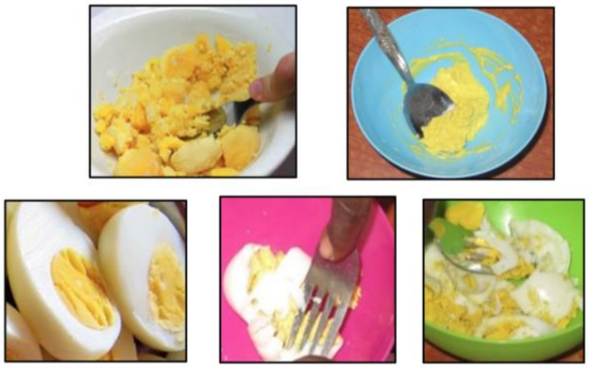

Figure 5. illustrates how to prepare the egg for the child.

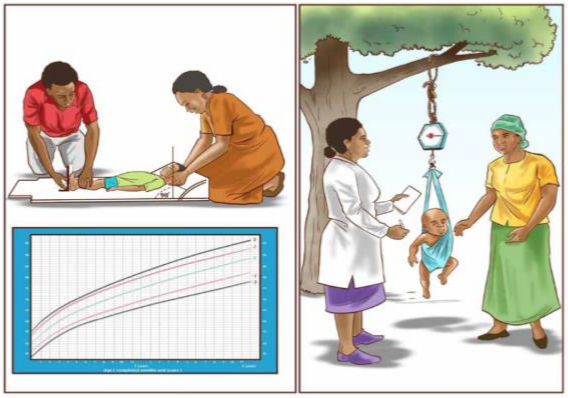

Credits: Millennium Village Project

Figure 6: Illustrates the importance of monitoring a child's health and growth.

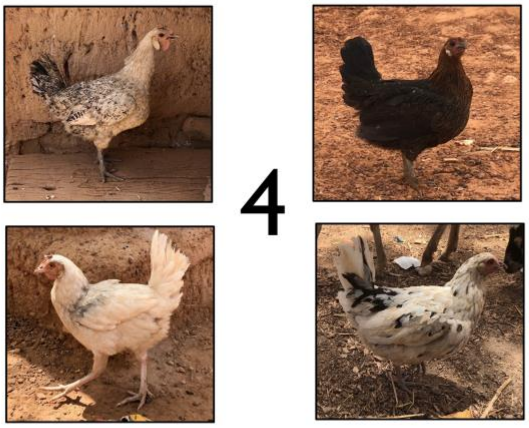

Figure 7. Illustrates that four chickens are recommend to produce enough eggs to feed a child an egg a day.

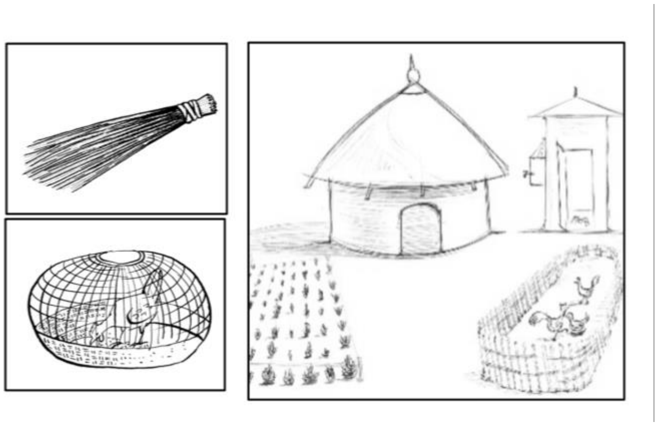

Figure 8. Illustrates the importance of keeping livestock in a separate area than the home environment as well as keeping the home environment clean and free of animal waste in efforts to prevent diseases caused by livestock.

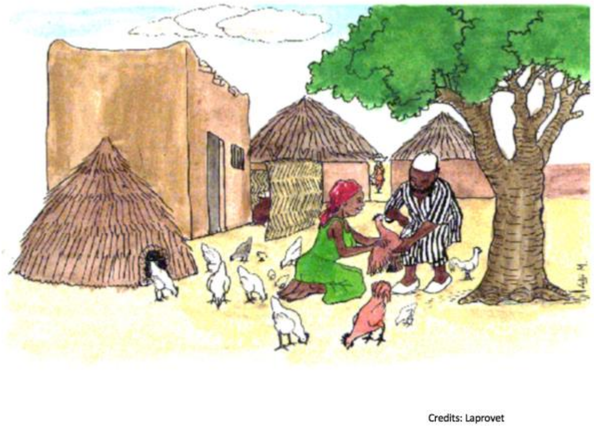


Figure 9. Illustrates the importance of vaccinating poultry to prevent disease and death among livestock.
